# Role of myeloid-derived suppressor cells in chronic brucellosis

**DOI:** 10.3389/fcimb.2024.1347883

**Published:** 2024-01-30

**Authors:** Shuiping Hou, Fandong Kong, Xintong Li, Yanwen Xu, Shouyi Chen, Sheng Zhang, Ling Zhang, Tingting Li, Yongshui Fu, Chengyao Li, Wenjing Wang

**Affiliations:** ^1^ Department of Transfusion Medicine, School of Laboratory Medicine and Biotechnology, Southern Medical University, Guangzhou, China; ^2^ Department of Microbiology, Guangzhou Center for Disease Control and Prevention (CDC), Guangzhou, China; ^3^ Department of Medical Administration, He Xian Memorial Affiliated Hospital of Southern Medical University, Guangzhou, China; ^4^ Department of Blood Components, Guangzhou Blood Center, Guangzhou, China; ^5^ Department of Obstetrics, He Xian Memorial Affiliated Hospital of Southern Medical University, Guangzhou, China; ^6^ Department of Parasitic Disease and Endemic Disease Control and Prevention, Guangzhou Center for Disease Control and Prevention (CDC), Guangzhou, China; ^7^ Administration Office, Baoan Central Blood Station, Shenzhen, China; ^8^ Clinical Transfusion Institute, Guangzhou Blood Center, Guangzhou, China

**Keywords:** brucellosis, chronic infection, MDSCs, immunosuppression, Arginase-1

## Abstract

**Introduction:**

Human brucellosis, a *Brucella* infection caused most common zoonosis in the world, remains a serious public health burden in China. *Brucella* chronic infection always causes immunosuppressive status and results in severe organ or tissue damages. The aim of this work was to study the role of the myeloid-derived suppressor cells (MDSCs) in human chronic brucellosis.

**Methods:**

Fifty cases of chronic brucellosis and 40 healthy individual controls were enrolled in this study. We analyzed the frequency and subsets of MDSCs in PBMC between the chronic brucellosis and healthy control groups by flow cytometry. Furthermore, we also measured the inflammatory-related cytokines in serum samples and the MDSCs inhibition ability to the proliferation of T cells *in vitro*.

**Results:**

We found that the frequency of MDSCs in peripheral blood and the level of IL-6 and IL-10 Th2 cytokines and Arginase-1 were significantly increased in chronic brucellosis patients. In addition, we also found that the T cell function was suppressed in vitro by co-culturing with MDSCs from brucellosis patients.

**Conclusion:**

Our study described an increase of immunosuppressive MDSCs in peripheral blood of chronic brucellosis patients. These results contribute to the understanding of *Brucella* persistent infection, which may provide an insight for effective treatment of chronic brucellosis patients in clinical practice.

## 1 Introduction

Human brucellosis is one of the most common bacterial zoonosis worldwide with over half a million new cases annually, and an incidence rate in some countries exceeding ten cases per million of population (Pappas et al., 2006). The disease is often transmitted from infected animals or unpasteurized animal products to humans, and the human to human transmission has also been described by vertical or sexual routes (Tuon et al., 2017). In humans, the brucellosis may cause many symptoms varying from mild flu-like to severe complications such as the central and peripheral nervous system, gastrointestinal, genitourinary, musculoskeletal, and cardiovascular systems (Franco et al., 2007; Galinska and Zagorski, 2013). Once infected, most cases firstly enter the acute phase of the disease. If without proper treatment, the acute phase may progress to chronic phase with relapse, development of persistent localized infection and non-specific syndrome, resembling the “chronic fatigue syndrome” (Corbel, [[NoYear]]).

The first line of defences against *Brucella* infection is innate immunity. Autophagy is one of the main elimination mechanisms to degrade intracellular pathogens. It involves the coordinated actions of immune cells like macrophages, immune molecules like pattern recognition receptors (PRRs), pathogen-associated molecular patterns (PAMPs), and complement systems (Yang et al., 2024). Although almost 90% of internalized *Brucellae* are killed with the first stage of infection, some evade the cell-mediated immunity by stealthy mechanisms which enable them survive along with the immune responses and replicate within intracellular niches (Amjadi et al., 2019). The previous research showed that chronic brucellosis reduced lymphocyte proliferation and Th1 cytokine secretion such as IFN-γ, but it enhanced TGF-β production compared to acute brucellosis (Ghaznavi et al., 2017).

Myeloid-derived suppressor cells (MDSCs) are a highly heterogeneous cell population of myeloid origin with potent immunosuppressive activity that comprises myeloid progenitor cells, immature macrophages, immature granulocytes and immature DCs (Gabrilovich and Nagaraj, 2009). The immature myeloid cells differentiate into mature macrophages, granulocytes and DCs under healthy conditions. However, in pathological conditions such as cancer, trauma and some infectious diseases, their maturation process was blocked, the PAMPs (LPS, flagellin, viral proteins) and other soluble cytokine factors activated MDSCs expansion, and a large number of MDSCs were released into bloodstream (Penaloza et al., 2019). Meanwhile, the immune suppressive factors such as arginase-1, inducible nitric oxide synthase (iNOS) and reactive oxygen species (ROS) were also increased (Rodriguez et al., 2009; Tacke et al., 2012; Wei et al., 2015).

Many studies focused on the role of MDSCs in tumor progression, accumulating in the micro-environment to escape the immune response by suppressing the activity and proliferation of T cells (Rodriguez et al., 2004; Qu et al., 2016). MDSCs have also caused attention in infectious diseases such as Mycobacterium tuberculosis, HIV and Aspergillus fumigatus infections because MDSCs from these patients can prevent the immune system from mounting effective immune response, resulting pathogen persistence and chronic infection (Medina and Hartl, 2018). Nevertheless, the role of MDSCs in chronic brucellosis has never been reported. In this study, we analyzed the frequency of MDSCs in peripheral blood of chronic brucellosis patients using HLA-DR^-/low^CD11b^+^ CD33^+^ as the MDSC markers and furtherly described the mechanism of MDSCs inducing immune suppression in the patients.

## 2 Methods

### 2.1 Patients and healthy donors

The individuals who were risk for exposure to *Brucellae* were tested for brucellosis antibody by Rose Bengal Test (RBT) and confirmed by Standard Agglutination Test (SAT) (Tsingtao Sinova-HK Biotechnology, China) in Guangzhou center for disease control and prevention (CDC) of China from January 2018 to December 2021. Fifty cases of chronic brucellosis patients (age: 21-74 years; mean age: 45.5 ± 13.24 years; 37 males and 13 females), and 40 healthy individual controls (age: 27-64 years; mean age: 41.43 ± 9.14 years; 30 males and 10 females) were enrolled, respectively. This study was approved by the Ethics Committee of the Guangzhou CDC, China.

### 2.2 Inclusion criteria

Patients who met the diagnostic criteria for brucellosis guidelines “Diagnosis for brucellosis WS269-2019” issued by National Health Commission of the People’s Republic of China in 2019 were included. The symptoms of the disease lasted for more than six months without recovery, and the serological reaction was positive.

### 2.3 Exclusion criteria

The exclusion criteria were as follows: (1) patients co-infected with HIV or other infectious diseases; (2) patients with a history of tumor or taking drugs for chemotherapy; (3) patients with lupus erythematosus and other autoimmune diseases; (4) patients who used immunosuppressive drugs, corticosteroids, and immunomodulators for a long time.

### 2.4 Isolation of peripheral blood mononuclear cells

Venous blood samples were collected from all subjects into EDTA-2k tubes and centrifuged at a speed of 3500 rpm for 10 min. Plasma samples were stored at -80°C. Buffy coat was diluted with D-PBS(Invitrogen, USA) at the ratio of 1:1 (v:v) and mixed gently. The suspension was transferred to a Corning tube containing 5ml of Ficol (Histopaque-1077; Sigma Chemical Co). Gradients were then subjected to centrifugation (2000rpm for 20 min at room temperature) and peripheral blood mononuclear cells (PBMCs) were recovered from the D-PBS/histopaque interface. PBMCs were washed in D-PBS at 1500rpm for 10min and then resuspended in D-PBS at a density of 1×10^7^cells/ml.

### 2.5 Flow cytometry

The frequency and subsets of MDSCs in PBMCs were determined by flow cytometry with the following antibodies to HLA-Dr, CD11b, CD33, CD14 and CD15. Flow cytometry was performed by Beckman Coulter (Navios, USA), and the data was analyzed by FlowJO (V10.8.0, USA). The isotype-matched antibodies were used as controls.

### 2.6 Isolation of MDSCs

MDSCs were isolated from PBMCs by HLA-Dr negative selection and followed by CD33 positive selection, using anti-HLA-Dr and anti-CD33 antibody-coated magnetic beads according to the manufacturer’s instructions (Miltenyi Biotech, Germany). Briefly, 1.0×10^7^ cells in 80μl of PBS buffer were incubated with 20μl of HLA-Dr MicroBead Cocktail. The sample was mixed well and incubated at 2-8°C for 10min. The mixture was transferred to the separation columns (MACS MS column; Miltenyi Biotech). The effluent was collected and incubated with 20μl of CD33^+^ MicroBead Cocktail. The cells were washed with 1ml of PBS buffer and the HLA-Dr^-^ CD33^+^ cells were collected. Purity of the separated cells was >90% by flow cytometry. CD3^+^ T cells were also isolated as stated above using Pan T magnetic beads (Miltenyi Biotech). CD4^+^ T cells and CD8^+^ T cells were isolated from PBMCs of healthy controls using CD4^+^ and CD8^+^ MicroBeads respectively (Miltenyi Biotech, Germany).

### 2.7 Measurement of T-cell suppression by T cell proliferation assay and of cytokine detection

The suppressive capacity of MDSCs was determined by the mixed allogeneic lymphocyte reaction. HLA-Dr^-^CD33^+^cells were isolated from the brucellosis patients as described. The responder CD4^+^/CD8^+^ T cells from healthy controls were stained with 1.5μM CFSE (Sigma, USA) according to the manufacture’s protocols. In a standard way, 10,000 CD4^+^/CD8^+^ T cells in RPMI1640 medium were seeded in 96 wells plates, and then 0, 10,000, 5000, 2500 HLA-Dr-CD33^+^ cells were added to the T cells at ratio of 0:1, 1:1, 1:2, 1:4 (MDSCs:T cells). Anti-CD3/CD28 antibodies coated beads (Gibco, USA) were also added and served as stimuli. The cell culture medium was supplemented with 10% FBS (Gibco), penicillin-streptomycin 100IU/ml (Gibco) and human recombinant IL-2 (Gibco). After incubation in humid atmosphere at 37°C and 5% CO_2_ for 72 hours, T cells were harvested and analyzed by flow cytometry. IFN-γ in the co-cultured supernatants was measured using an ELISA kit (Invitrogen, USA), according to the manufacturer’s instructions.

### 2.8 Enzyme-linked immunosorbent assay

All the serum or plasma samples of the participants and the supernatants of mixed lymphocyte reactions were stored at -80°C. The concentration of IL-2, IL-6, IL-10, TNF-α, TGF-β, IFN-γ, Arginase-1 and iNOS were determined by the ELISA kits according to the manufacture’s protocols (IL-2 and IL-10: Dakewei Biotec, China; IL-6, TNF-α and TGF-β: Biolegend, USA; IFN-γ and Arginase-1: Invitrogen, USA; iNOS: Biovision, USA. The optical density (OD) value of the sample reaction was measured at the 450nm by the microplate reader (Bio-Rad, USA).

### 2.9 Quantitative polymerase chain reaction (RT-qPCR)

HLA-Dr^-^CD33^+^ cells were isolated as described previously. The total RNA was extracted from MDSCs from chronic brucellosis patients and healthy individual controls by using Trizol (RNAsimple Total RNA Kit, Tiangen, China). The concentration and the purity of RNA were measured by Qubit. RT-qPCR was performed with SYBR One Step RT-qPCR kit (FastKing Real Time One Step RT-qPCR, Tiangen, China) using β-actin gene as a control for normalization of the data. Gene relative expression was normalized to β-actin gene expression using the 2^−ΔΔCT^ method (Livak and Schmittgen, 2001).

### 2.10 Measurement of arginase activity

MDSCs and T cells were isolated as described above. Cells were cultured for 72h in RPMI medium supplemented with 10% FBS (Invitrogen), penicillin-streptomycin 100IU/ml (Invitrogen). Supernatant was collected and stored at -80°C. The arginase activity was measured by Arginase Activity Colorimetric Assay Kit according to the instruction (BioVision, USA).

### 2.11 Statistical analysis

Difference in normally distributed variables and non-normally distributed variables between the two groups were tested by *t*-test and Wilcoxon test, respectively. Statistical analysis was performed using GraphPad Prism 8 (GraphPad Software Company, USA). *P*<0.05 was considered significant.

## 3 Results

### 3.1 Frequency of MDSCs in PBMCs from chronic brucellosis patients

**Figure 1:**
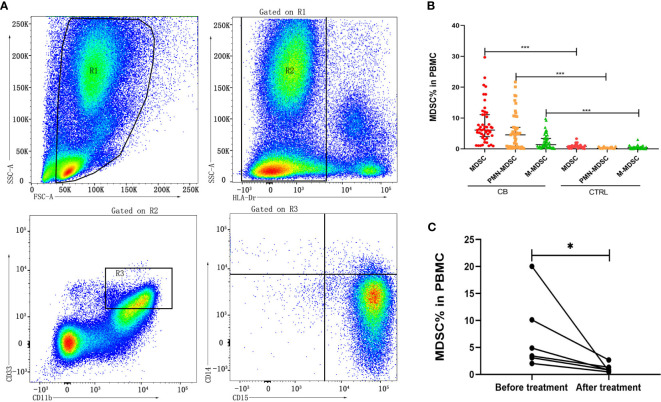
Frequency of MDSCs in PBMCs from chronic brucellosis patients and healthy controls. **(A)** Gating strategy of flow cytometry analysis for MDSCs in PBMCs. **(B)** The frequency MDSCs, M-MDSCs and PMN-MDSCs in PBMCs from 50 chronic brucellosis patients (CB) compared with that from 40 healthy controls (CTRL). **(C)** The frequency of MDSCs in PBMCs from six followed-up chronic brucellosis patients before and after treatment. *P <0.05, ***P <0.001.

Human MDSCs can be divided into two major subsets of monocytic (M)-MDSC (HLA-Dr^low/-^/CD11b^+^/CD33^+^/CD14^+^/CD15^-^) and polymorphonuclear (PMN)-MDSC (HLA-Dr^low/-^/CD11b^+^/CD33^+^/CD14^-^/CD15^+^) according to the different phenotype (Sade-Feldman et al., 2016). The frequencies of these two subsets of MDSCs in PBMCs from brucellosis patients were calculated in comparison with the frequencies from healthy control cohorts (**Figure 1A**). The frequencies of MDSCs, M-MDSCs and PMN-MDSCs were significantly increased in chronic brucellosis patients than those in healthy controls (*P*<0.001; **Figure 1B**). In six followed-up patients who received antibiotics and symptomatic treatment for 2-3 months, the frequency of MDSCs in PBMCs was detected significantly lower than that before treatment (*P*<0.05; **Figure 1C**).

### 3.2 Higher immunosuppressive cytokines in chronic brucellosis patients

**Figure 2:**
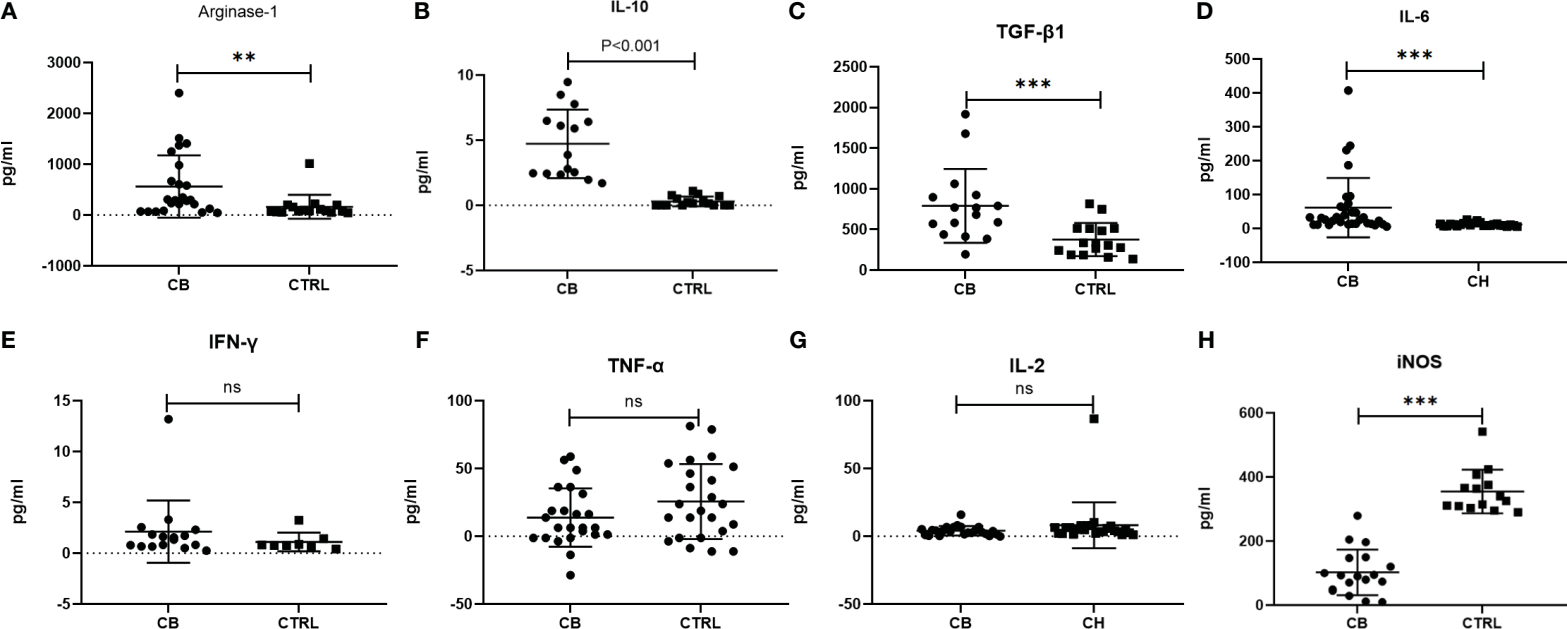
Measurement of serum inflammatory-related cytokines from chronic brucellosis patients (CB) and healthy individuals (CTRL) by ELISA. **(A)** Arginae-1 (CB, n=24; CTRL, n=16); **(B)** IL-10 (CB, n=15; CTRL, n=16); **(C)** TGF-β (CB, n=16; CTRL, n=16); **(D)** IL-6 (CB, n=32; CTRL, n=24); **(E)** IFN-γ (CB, n=16; CTRL, n=8); **(F)** TNF-α (CB, n=24; CTRL, n=24); **(G)** IL-2 (CB, n=24; CTRL, n=24); **(H)** iNOS (CB, n=18; CTRL, n=14). **P<0.01; ***P<0.001; ns, no significance.

The inflammatory-related cytokines (Arginase-1, iNOS, IL-10, TGF-β1, IL-6, IFN-γ, TNF-α and IL-2) were measured in serum samples by ELISA from chronic brucellosis and healthy control groups (**Figure 2**). The level of Arginase-1, IL-10, TGF-β1 and IL-6 was significantly higher in chronic brucellosis group than that in healthy control group (*P*<0.01; **Figures 2A-D**), while the level of IFN-γ, TNF-α and IL-2 varied insignificantly between two groups (*P*>0.05; **Figures 2E-G**), suggesting that the higher immunosuppression cytokines in chronic brucellosis patients than that in heathy individuals. The level of iNOS was statistically lower in brucellosis patients than that in healthy individuals (*P*<0.001; **Figure 2H**).

### 3.3 Chronic brucellosis patients’ MDSCs suppress T cell proliferation *in vitro*

**Figure 3:**
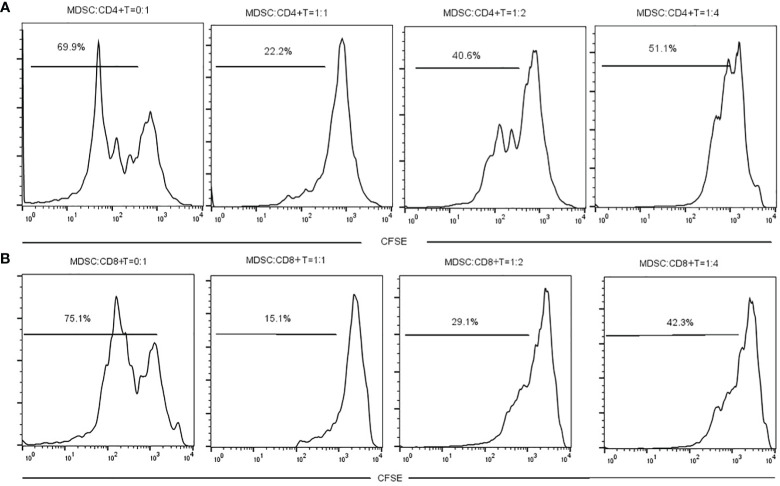
The measurement of CD4^+^ or CD8^+^ T cell proliferation with or without MDSCs co-culture for 3 days. **(A)** MDSCs were co-cultured with CD4^+^ T cells at a ratio of 0:1, 1:1, 1:2 or 1:4. **(B)** MDSCs were co-cultured with CD8^+^ T cells at a ratio of 0:1, 1:1, 1:2 or 1:4.

**Figure 4:**
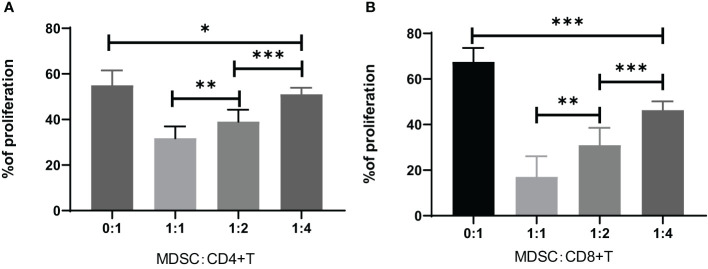
Inhibition of CD4^+^ or CD8^+^ T cell proliferation by co-culturing for 3 days with MDSCs at the ratio of 0:1, 1:1, 1:2 or 1:4. **(A)** The percentage of CD4^+^ T cell proliferation co-cultured with MDSCs. 0:1, n=12; 1:1, n=7; 1:2, n=15; 1:4, n=16. **(B)** The percentage of CD8^+^ T cell proliferation co-cultured with MDSCs. 0:1, n=10; 1:1, n=10; 1:2, n=7; 1:4, n=7. *P<0.05; **P<0.01; ***P<0.001.

The MDSCs were isolated from chronic brucellosis patients and co-cultured with CD4^+^/CD8^+^ T cells from healthy controls at the ratio of 0:1, 1:1, 1:2 and 1:4 for 72 hours (**Figure 3**). In the culturing without MDSCs (a ratio of 0:1), both CD4^+^ and CD8^+^ T cells showed a strong proliferation ability (**Figure 3**), while in the co-culturing with MDSCs at the ratio of 1:1, 1:2 or 1:4, T cell proliferations presented an inhibition pattern clearly depending on the ratio of MDSCs than T cells (*P*<0.01; **Figure 4**).

**Figure 5:**
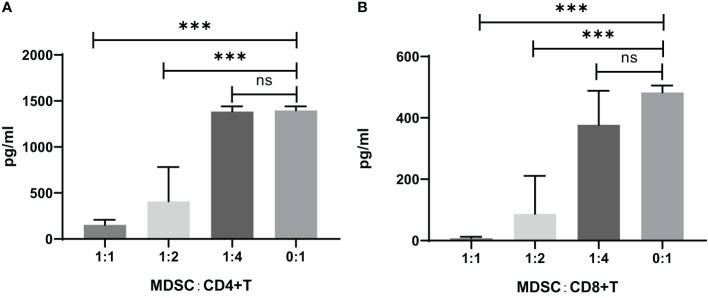
IFN-γ concentration in the supernatants of CD4^+^ or CD8^+^ T cell co-cultures with MDSCs *in vitro*. **(A)** CD4^+^ T cells co-cultivated with MDSCs. 0:1, n=8; 1:1, n=8; 1:2, n=3; 1:4, n=13. **(B)** CD8^+^ T cells co-cultivated with MDSCs. 0:1, n=2; 1:1, n=12; 1:2, n=12; 1:4, n=10. ns, no significance; ***P<0.001.

The IFN-γ concentration in the CD4^+^ or CD8^+^ T cell co-cultured supernatants with MDSCs was measured by ELISA, which showed that the IFN-γ concentration was increased by the decreased ratio of MDSCs in the co-culture of CD4^+^ or CD8^+^ cells (**Figure 5**).

### 3.4 Chronic brucellosis patients’ MDSCs release arginase-1 and iNOS into the circulation of the patients and the co-cultures

**Figure 6:**
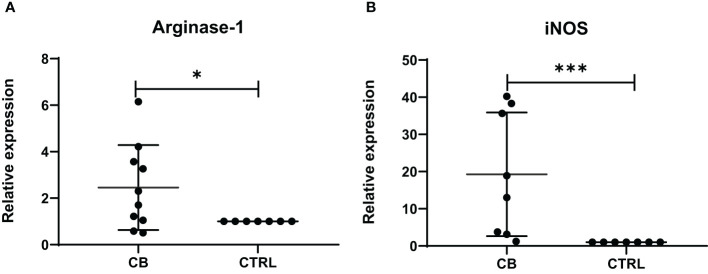
The relative expression of Arginase-1 and iNOS mRNA transcripts in MDSCs from chronic brucellosis patients or healthy individuals. **(A)** Arginase-1; **(B)** iNOS. *P<0.05; ***P<0.001.

**Figure 7:**
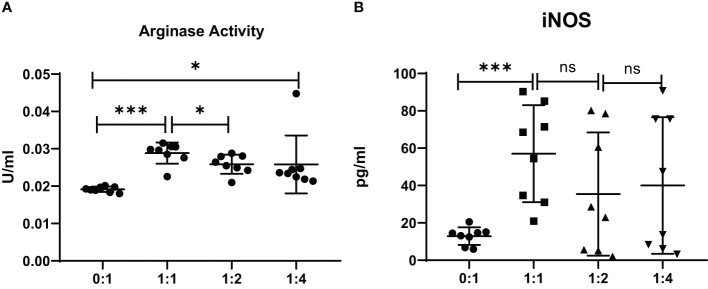
The activity of arginase and iNOS in the supernatants of co-cultures from MDSCs and T cells at various ratios (MDSCs:T cells). MDSCs were isolated from 8 chronic brucellosis patients or healthy individuals, respectively. **(A)** Measurement of arginase activity (U/ml). **(B)** Measurement of iNOS concentration (pg/ml). *P<0.05, ***P<0.001, ns, no significance.

The level of mRNA transcripts of Arginase-1 and iNOS were detected higher *in vitro* in MDSCs from brucellosis patients than that from healthy controls (**Figure 6**). Furthermore, the activity of arginase and iNOS in the co-culture supernatants of MDSCs and CD3^+^ T cells at a different ratio was tested. When brucellosis patients’ MDSCs were not added to T cell cultures, the arginase activity and iNOS releases were lower in the supernatants of T cell cultures (**Figure 7**). When patient’s MDSCs were added to co-cultivate with T cells at a ratio of 1:1, the level of Arginase activity (**Figure 7A**) and iNOS releases (**Figure 7B**) was significantly increased, of which the level of these two cytokines varied according to the ratio of MDSCs than T cells. The data suggested that MDSCs promoted releases of Arginase and iNOS.

## 4 Discussion

MDSCs were studied mostly in various cancers, which appeared to increase in the blood of cancer patients (Gabrilovich and Nagaraj, 2009). MDSCs were also found to play an immune effect in acute or chronic infectious diseases such as *Staphylococcus aureus*, *Hepatitis B* and *Candida albicans* (Mencacci et al., 2002; Skabytska et al., 2014; Li et al., 2020). In this study, we showed an increasing frequency of MDSCs in peripheral blood of chronic brucellosis patients (**Figure 1B**), which might be associated with an immunosuppressive status of chronic brucellosis patients (Groth et al., 2019), who had the higher level of serum IL-10 and TGF-β compared to healthy individuals (**Figures 2B, C**).

MDSCs are absent or rare in the peripheral blood of healthy people, constituting 0.5% of PBMCs (Almand et al., 2001; Rieber et al., 2013). When cancer, inflammation and infection occur in hosts, the number of MDSCs expands in microenvironment, tissue or peripheral blood (Groth et al., 2019). On the one hand, MDSCs can benefit the host by reducing the immune-mediated response that cause damage to the host, such as in the polymicrobial sepsis, and also MDSCs can prevent the sepsis-associated mortality (Sander et al., 2010). On the other hand, MDSCs have strong ability to suppress effective T cell immune response, resulting to pathogen persistence and chronic infection (Sander et al., 2010). It has been reported that different bacteria induce distinct MDSC subsets: the proportion of PMN-MDSCs always increase in sepsis patients infected by Gram-positive bacteria, while M-MDSCs are always induced by Gram-negative bacteria (Janols et al., 2014). In our study, two subsets of PMN-MDSCs and M-MDSC were all accumulated in the blood of chronic brucellosis patients and significant higher than those in healthy controls, and the proportion of PMN-MDSCs is larger than that of M-MDSCs (**Figure 1B**). *Brucella* persistent infection is often considered by the “stealth strategy” to evade the innate and adaptive immune response (Ahmed et al., 2016), while our study suggests that the MDSCs may be a key factor contributing to *Brucella* persistent infection.

Though the pathogenesis of *Brucella* infection has not been fully understood, it is presumed that *Brucella* can evade the immune system and cause T lymphocyte disorder, and lead chronic infection (Zheng et al., 2019). Many reports show that T lymphocyte subsets and immune responses between the acute and chronic phases of brucellosis are often different (Rafiei et al., 2006; Skendros et al., 2011; Ghaznavi et al., 2017). In our study, we found the Th1 cytokines such as IL-2, TNF-α and IFN-γ, have no difference between the chronic brucellosis and the healthy control groups, but the Th2 cytokines such as IL-10 and IL-6 in brucellosis group are significantly higher than these in control group (*P*<0.001). These results suggest that chronic brucellosis presents an immunosuppressive status. MDSCs have an immunosuppressive function via suppressing the proliferation and activity of T cells (Rieber et al., 2013; Li et al., 2015; Onyilagha et al., 2018; Wang et al., 2019). In this study, we examined T cell function by co-culturing with MDSCs from peripheral blood of chronic brucellosis patients, showing that CD4^+^ and CD8^+^ T cell proliferation and their IFN-γ expression were largely inhibited by MDSCs (*P*<0.001; **Figures 3**-**5**).

Besides MDSCs induced T cell dysfunction, several studies have also reported that MDSCs can trigger T cell apoptosis in tumor microenvironment as the relevant mechanism of tumoral immune resistance (Zhu et al., 2017; Horton et al., 2018). Researchers found that MDSCs in TiRP tumors expressed high levels of FasL and caused T cell apoptosis by Fas-FasL apoptotic pathway (Zhu et al., 2019). As a result, with the expansion of MDSCs in chronic brucellosis, it might trigger T cell apoptosis by fasL-mediated attack *in vivo*, which would strongly impair T cell function and cause *Brucella* persistent infection in patients. However, this hypothesis needs to be demonstrated furtherly.

MDSCs are divided into two major subsets of PMN and M-MDSCs based on their phenotypic and morphological features (Bronte et al., 2016). We found that chronic brucellosis patients had higher frequencies of PMN-MDSCs and M-MDSCs compared with healthy individuals (*P*<0.001; **Figure 1B**), while the proportion of PMN-MDSCs is higher than that of M-MDSCs. Both PMN-MDSCs and M-MDSCs can express high level of Arginase-1, which converts L-arginine into urea and L-ornithine, and then the shortage of L-arginine inhibits T cell proliferation by decreasing the expression of CD3 ζ-chain and preventing upregulation of the expression of cell cycle regulators (Gabrilovich and Nagaraj, 2009). In addition, M-MDSCs increase STAT1 and iNOS expression and NO level but not ROS production, which inhibits the Janus kinase 3, STAT5 and MHC class II expression and induces T cell apoptosis (Movahedi et al., 2008), while PMN-MDSCs produce high level of ROS which induces post-translational modification of T-cell receptors (Gabrilovich and Nagaraj, 2009). PMN-MDSCs and M-MDSCs also utilize different mechanism of immune suppression: PMN-MDSCs are primarily in antigen-specific manner, while M-MDSCs suppress T cells both in antigen-specific and non-specific manners (Gabrilovich, 2017). In our study, the activity of arginase and iNOS was detected significantly increased when MDSCs were added to co-culture with T cells (*P*<0.001; **Figures 7A, B**), which might be an additional factor to impair T cell function through above mechanisms in chronic brucellosis patients.

In summary, this study described an increase of immunosuppressive MDSCs in peripheral blood of chronic brucellosis patients, which might contribute to *Brucella* persistent infection.

## Data availability statement

The raw data supporting the conclusions of this article will be made available by the authors, without undue reservation.

## Ethics statement

The studies involving humans were approved by Ethics Committee of the Guangzhou CDC. The studies were conducted in accordance with the local legislation and institutional requirements. The ethics committee/institutional review board waived the requirement of written informed consent for participation from the participants or the participants’ legal guardians/next of kin because The sample donors had signed an informed consent form, agreeing that the donated sample and related information can be used for all medical research.

## Author contributions

SH: Methodology, Writing – original draft. FK: Methodology, Writing – review & editing. XL: Methodology, Writing – review & editing. YX: Methodology, Writing – review & editing. SC: Formal Analysis, Writing – review & editing. SZ: Formal Analysis, Writing – review & editing. LZ: Formal Analysis, Writing – review & editing. TL: Formal Analysis, Writing – review & editing. YF: Project administration, Writing – review & editing. CL: Writing – review & editing, Writing – original draft. WW: Writing – review & editing.
